# Cafedrine/Theodrenaline (20:1) Is an Established Alternative for the Management of Arterial Hypotension in Germany—a Review Based on a Systematic Literature Search

**DOI:** 10.3389/fphar.2017.00068

**Published:** 2017-02-21

**Authors:** Berthold Bein, Torsten Christ, Leopold H. J. Eberhart

**Affiliations:** ^1^Department of Anesthesiology and Intensive Care Medicine, Asklepios Hospital St. GeorgHamburg, Germany; ^2^Department of Experimental Pharmacology and Toxicology, University Medical Centre Hamburg-EppendorfHamburg, Germany; ^3^Germany and German Centre for Cardiovascular Research, University Medical Centre Hamburg-EppendorfHamburg, Germany; ^4^Department of Anesthesiology and Intensive Care Medicine, Philipps-University MarburgMarburg, Germany

**Keywords:** cafedrine/theodrenaline drug combination, spinal hypotension: treatment, arterial hypotension: treatment, heart frequency, inotropy, obstetric anesthesia

## Abstract

A 20:1 combination of cafedrine:theodrenaline (Akrinor®) is widely used in Germany for the treatment of hypotensive states during anesthesia and in emergency medicine. Although this drug formulation has been available since 1963, there are few studies relating to its use and many of the data are only available in German. In this article, we summarize the available data and propose mechanisms for the effects of cafedrine/theodrenaline on cardiac muscle cells and vascular smooth muscle cells. Cafedrine/theodrenaline leads to a rapid increase in mean arterial pressure that is characterized by increased cardiac preload, stroke volume, and cardiac output. Systemic vascular resistance and heart rate remain mostly unchanged. Factors which impact the effects of cafedrine/theodrenaline are gender, high arterial pressure at baseline, use of β-blockers, and heart failure. Importantly, the drug is frequently used in obstetric anesthesia without detrimental effects on umbilical cord pH or APGAR score.

## Introduction

Hypotension occurs frequently after induction of anesthesia or in emergency medicine. Some organs are especially susceptible to hypotension (Weyland and Grüne, 2013): the brain, which has a high oxygen demand and a variable autoregulatory threshold. The kidney and the heart need adequate perfusion pressure for ultrafiltration (Walsh et al., 2013; Weyland and Grüne, 2013) and to maintain coronary blood flow (Walsh et al., 2013; Weyland and Grüne, 2013). In hypotensive patients, timely restoration of blood pressure is important to avoid end-organ damage (Walsh et al., 2013).

In Germany, a combination of cafedrine (covalently linked norephedrine and theophylline) and theodrenaline (covalently linked noradrenaline and theophylline) called Akrinor® is used for the treatment of hypotension in adults that occurs during emergency situations, general anesthesia, and regional anesthesia, especially during cesarean sections (Koch and Knoth, 2006). Hypotensive states during general anesthesia or following spinal or epidural anesthesia are the most common situations in which this drug is administered (Koch and Knoth, 2006). Cafedrine/theodrenaline has been widely used in Germany since 1963. In 2005, AWD.pharma GmbH & Co. KG (the company which owned the rights to the drug at the time) decided to take cafedrine/theodrenaline off the market because the requirements for subsequent approval could not be met within the required timeframe (Radke, 2005; Koch and Wenzel, 2006). However, because there was no alternative product with comparable pharmacodynamic characteristics on the German market at that time, the federal authority granted provisional approval after consultation with the German Society of Anesthesiology and Intensive Care (Deutsche Gesellschaft für Anästhesiologie und Intensivmedizin; DGAI; Koch and Wenzel, 2006; Radke, 2006). In 2012, cafedrine/theodrenaline received regular approval by German federal authorities after meeting the approval requirements (ratiopharm GmbH, 2013).

While hypotensive states in other countries are often treated with ephedrine and phenylephrine (Lonjaret et al., 2014), German anesthesiologists often use cafedrine/theodrenaline and have gained a lot of practical experience with the substance. 86.2% of German hospitals use cafedrine/theodrenaline for the treatment of hypotension during regional anesthesia for cesarean section (Marcus et al., 2011).

However, few data relating to its use have been published so far and the majority of existing publications are only available in German. Thus, to give the entire scientific community access to these data, we aim to summarize the current state of knowledge by providing a comprehensive English-language overview. We consider this of particular importance for on-going studies in non-German-speaking countries.

Cafedrine/theodrenaline may have advantages over other vasopressor drugs. For example, it can be administered via bolus while catecholamines normally need to be diluted and administered via syringe pumps. Bolus injection is faster, which may be beneficial in emergency situations, plus it is more cost efficient with respect to the disposables.

The combination of cafedrine and theodrenaline is unique and the substances complement one another. Sole administration of theodrenaline increases vascular resistance, while cafedrine exhibits an inotropic effect (Sternitzke et al., 1984). By administering the two substances in combination the optimal ratio of cafedrine and theodrenaline can be used. Sakai et al. tested different ratios in dogs and rats and concluded that 20:1 is the ideal ratio for both a rapid onset and a long-lasting hypertensive effect (Sakai et al., 1972a).

## Methods

MEDLINE®, BIOSIS Previews®, Embase®, and SciSearch® were systematically searched using the following terms: akrinor OR acrinor OR praxinor OR H-835 OR H835 OR “cafedrine—theodrenaline” OR (cafedrine AND theodrenaline). The German and English results were screened manually for relevance. Reference lists of internal reports provided by the manufacturer of the drug were screened for additional publications.

## Pharmacokinetics

The pharmacokinetics of cafedrine/theodrenaline have not been the subject of extensive research. Theodrenaline's pharmacokinetic properties have not been characterized due to this agent's lack of stability and the low doses administered. There are few data available for cafedrine. The initial plasma level of cafedrine after intravenous administration of 200 mg is 6 μg.ml^−1^. Cafedrine is metabolized to norephedrine and several minor metabolites, as summarized by Koch and Knoth (2006). However, nearly 90% of the administered norephedrine is excreted via the kidneys, mostly unchanged, within 24 h (Sinsheimer et al., 1973). Cafedrine has a half-life of 60 min following both oral and intravenous administration (summarized in Koch and Knoth, 2006). Data are hard to interpret since it is not known whether cleavage products are active or even more active than cafedrine itself; exact metabolites and their potency are unknown.

## Effects on α- and β-adrenergic receptors

**Figure 1:**
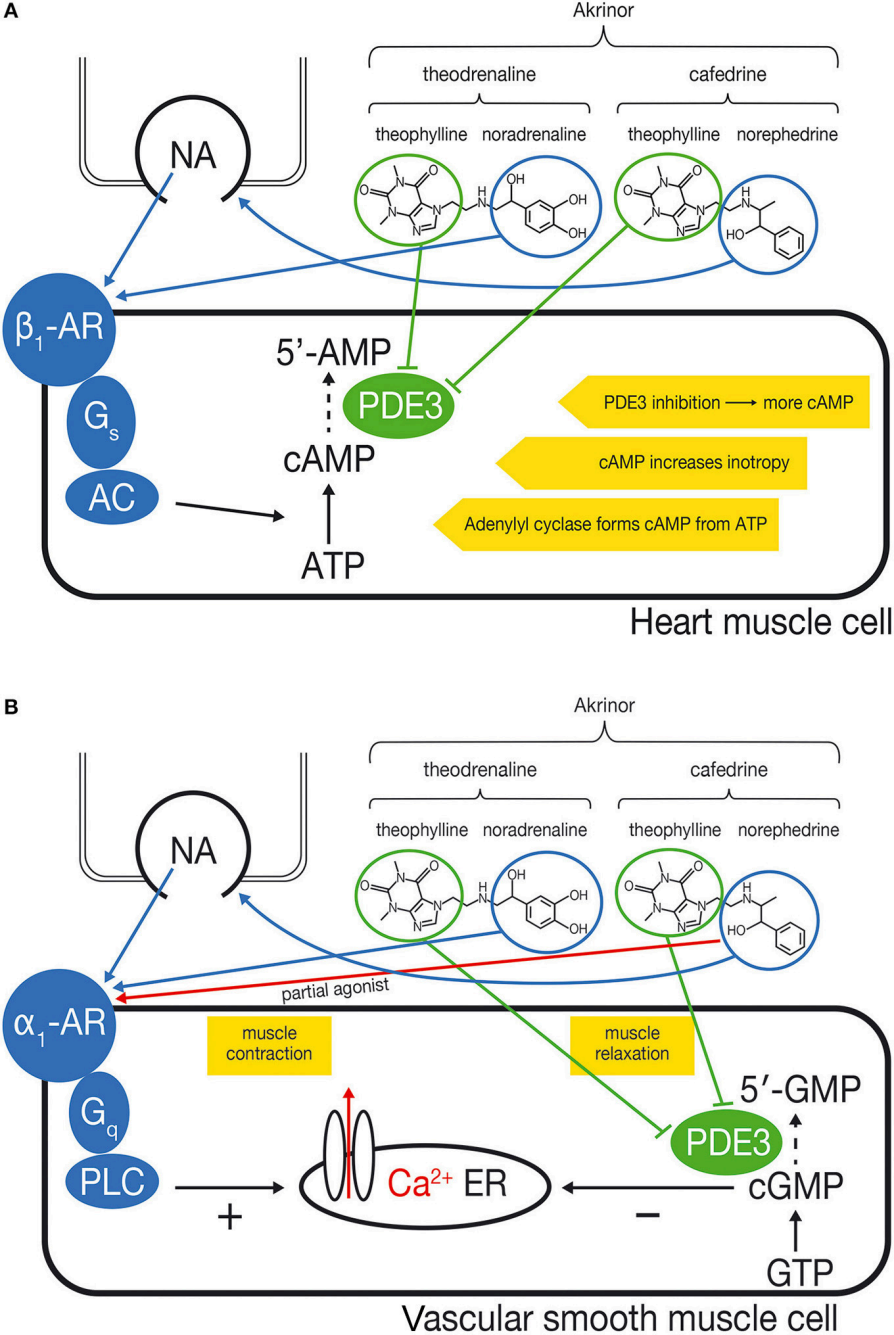
**(A)** Proposed mechanism of action of cafedrine/theodrenaline in cardiomyocytes: increased inotropy. Cafedrine/theodrenaline is a combination of norephedrine and theophylline (cafedrine) and noradrenaline and theophylline (theodrenaline). The norephedrine component releases noradrenaline from endogenous stores (nerve endings). The endogenously released noradrenaline and the noradrenaline component of theodrenaline activate the β_1_-adrenoceptor (β_1_-AR) in the heart muscle cell, which leads—via G_*s*_-proteins—to activation of adenylyl cyclase (AC). Adenylyl cyclase catalyzes the conversion of ATP to cAMP which increases inotropy. The theophylline component of cafedrine and theodrenaline is expected to inhibit the phosphodiesterases (PDEs) in an unselective manner. PDE3 is the most relevant PDE in human cardiac tissue. Inhibition of PDE slows degradation of cAMP and increases cAMP concentration, thereby reinforcing the β_1_-adrenoceptor stimulation. **(B)** Proposed mechanism of action of cafedrine/theodrenaline in vascular smooth muscle cells: contradictory effects. The noradrenaline component of theodrenaline activates the α_1_-adrenoceptor of the vascular smooth muscle cell. This leads—via G_q_-proteins—to activation of phospholipase C (PLC) which ultimately leads to release of Ca^2+^ from the endoplasmic reticulum (ER) into the cytosol, thereby initiating muscle contraction. The norephedrine component of cafedrine stimulates the release of endogenous noradrenaline but may also act as a partial agonist at the α_1_-adrenoceptor, thereby mediating vasoconstriction by itself but possibly reducing the effects of endogenous noradrenaline and of the noradrenaline component of theodrenaline. The positive effect on muscle contraction may be counteracted further by a vasodilatory effect of theophylline, which is thought to inhibit PDE3 and therefore the degradation of cGMP to 5′-GMP. This would result in accumulation of cGMP. cGMP inhibits release of Ca^2+^ into the cytosol, leading to relaxation of the muscle, thus counteracting α_1_-mediated vasoconstriction. The net effect of α_1_-mediated vasoconstriction and cGMP-mediated vasodilatation on the vascular muscle cell has not been described yet and may vary depending on the specific vessel and local distribution of α_1_-adrenoceptors.

Blood pressure is determined by cardiac output and systemic vascular resistance. The cardiac effect of cafedrine/theodrenaline is mediated by β-adrenoceptors, while the resistance of the blood vessels is largely controlled by α-receptors (Ahlquist, 1967). Possible mechanisms of action of cafedrine/theodrenaline on cardiac muscle cells and vascular smooth muscle cells are summarized in Figure 1.

The effects of cafedrine/theodrenaline on cardiac output are believed to be mediated via β-receptors. Cafedrine/theodrenaline has a positive inotropic effect in humans, and this can be abolished by administration of the non-selective β-adrenoceptor antagonist propranolol (Sternitzke et al., 1976, 1984). In anesthetized patients on β-blocker therapy, onset of theodrenaline/cafedrine's effect on blood pressure is delayed and the effect size is decreased (Heller et al., 2008, 2015).

The effect of cafedrine/theodrenaline on α-receptors is still under debate (Sakai et al., 1969; Sternitzke et al., 1984; Usichenko et al., 2006) and may differ with respect to vessel region and species. In porcine coronary arteries, cafedrine/theodrenaline induces vasoconstriction only when β-adrenoceptors are blocked. This vasoconstriction by cafedrine/theodrenaline can be abolished by the α_1_-adrenoceptor blocker prazosin (Usichenko et al., 2006). Theodrenaline alone shows an α-adrenergic peripheral vasoconstrictor effect in healthy subjects (Sternitzke et al., 1984). However, this effect is counteracted by the vasodilatory component of cafedrine, as shown in a study with healthy subjects (Sternitzke et al., 1984) and dogs (Sakai et al., 1969). Norephedrine, which is a part of cafedrine, has been shown to act as a partial agonist at α_1_-adrenoceptors in rats (Minneman et al., 1983). Partial agonists can activate receptors. However, maximum effects are clearly smaller than with the full, natural agonist. Therefore, partial agonists behave as competitive antagonists in the presence of the full agonist. At present it is unclear if norephedrine bound to theophylline shares partial agonistic activity of norephedrine. Therefore, partial agonistic activity on α_1_-adrenoceptors by norephedrine could mediate vasoconstriction by itself but may reduce vasoconstriction evoked from noradrenaline.

Another expected effect of cafedrine/theodrenaline is the inhibition of phosphodiesterase (PDE), since both drugs contain the non-selective PDE inhibitor theophylline (Sternitzke et al., 1984). PDE degrades second messengers such as cAMP and cGMP which mediate inotropy and vascular muscle relaxation, respectively (Maurice et al., 2014). Because cafedrine/theodrenaline is a 20:1 mixture of the two drugs, it is believed that the PDE-inhibiting effect of cafedrine/theodrenaline is primarily mediated by cafedrine. PDE inhibition is thought to affect both the heart and the blood vessels (Maurice et al., 2003), even though it has not yet been proven that cafedrine's and theodrenaline's theophylline component exhibit this effect when administered in combination with norephedrine (from cafedrine) and noradrenaline (from theodrenaline). PDE3 inhibition alone can increase inotropy, independently of catecholamines (Christ et al., 2006; Molenaar et al., 2013). On the other hand, inhibition of PDE blunts α_1_-adrenoceptor-mediated vasoconstriction, as demonstrated by experiments on internal mammary artery segments (He and Yang, 1996). It is unclear to what extent PDE inhibition accounts for the effects of cafedrine/theodrenaline.

In summary we hypothesize that cafedrine/theodrenaline acts predominantly via β-adrenoceptors in the heart, directly activating β_1_-adrenoceptors, and probably reinforcing the effects of β_1_-adrenoceptor stimulation via PDE inhibition. Effects on isolated vessels are difficult to predict due to the different pharmacodynamics of the individual constitutes of cafedrine/theodrenaline.

## Individual effects of cafedrine and theodrenaline

If theodrenaline is intravenously administered as a single drug to healthy subjects, this leads to an immediate, rapid increase in mean arterial blood pressure (MAP) by 28%; this effect decreases gradually up to 20 min after administration (Sternitzke et al., 1984).

On the other hand, if cafedrine is intravenously administered as a single drug to healthy subjects without arterial hypotension, the increase in blood pressure is delayed, but lasts longer (Sternitzke et al., 1984). The maximum effect of 200 mg cafedrine was noted after 20 min (Sternitzke et al., 1984). However, one *in vitro* study suggests that the pressor effect of cafedrine may exhibit tachyphylaxis (Daweke and Oberdorf, 1958), while an animal study showed that repeated oral administration is not associated with a reduction in the pressor effect of this agent (Sakai et al., 1972b).

## Effects of cafedrine and theodrenaline in combination

### Cardiovascular effects

A number of animal and clinical studies have been published on the cardiovascular effects on cafedrine/theodrenaline. These studies involve different patient populations, different designs, and different measured outcomes. We give an overview of the relevant study details in Supplemental Table 1 (Supplemental Digital Content).

Administration of both drugs to healthy subjects leads to an increase in cardiac preload, stroke volume, and cardiac output (Fischer and Weis, 1965; Schieffer et al., 1971; Sternitzke et al., 1984; Muller et al., 1985). Cafedrine/theodrenaline has a positive inotropic effect (Fischer and Weis, 1965; Schieffer et al., 1971; Sternitzke et al., 1975, 1984; Muller et al., 1985) and the heart rate in awake patients is mildly decreased (Fischer and Weis, 1965; Sternitzke et al., 1976). Conversely, a more recent study reported an increase in heart rate in patients under anesthesia after cafedrine/theodrenaline administration (Heller et al., 2008).

Cafedrine/theodrenaline shows a mostly unchanged systemic vascular resistance in healthy subjects. (Fischer and Weis, 1965; Sternitzke et al., 1984).

Theodrenaline leads to a rapid, peripheral vasoconstriction mediated via α-adrenoceptors. This may be due to the noradrenaline component of theodrenaline (Sternitzke et al., 1984). This effect may be in competition to the partial agonistic activity of cafedrine's norephedrine component on α-receptors: Norephedrine may act as a partial agonist at the α1-adrenoceptor (Minneman et al., 1983), thereby mediating vasoconstriction by itself but possibly reducing the effects of endogenous noradrenaline and of the noradrenaline component of theodrenaline.

Animal data show an increased coronary blood flow after cafedrine/theodrenaline administration, which provides an increased amount of oxygen (Hahn et al., 1985). Together with the moderate decrease in heart rate, this may compensate for the increased oxygen consumption which results from increased inotropy (Hahn et al., 1985; Koch and Knoth, 2006). Studies involving anesthetized dogs in cardiogenic shock showed a 40% increase in oxygen consumption after administration of cafedrine/theodrenaline, but also an increase in blood flow by 181% (Hahn et al., 1985). Another study involving nine healthy anesthetized dogs showed similar results (Schlepper and Witzleb, 1962). The authors of these studies concluded that cafedrine/theodrenaline may provide a sufficient oxygen supply due to this compensation (Schlepper and Witzleb, 1962; Hahn et al., 1985; Heller et al., 2008). This might be one explanation for the positive profile of cafedrine/theodrenaline in patients with myocardial infarction (Heller and Grosser, 1974; Koch and Knoth, 2006; Heller et al., 2008). It should be noted that in patients with myocardial injury and hypotension, the increase in blood pressure is substantially larger than in healthy subjects (18 mmHg vs. 49 mmHg after intramuscular administration; Schleusing and Bartsch, 1963).

### Patients undergoing general or regional anesthesia

The effects of cafedrine/theodrenaline were investigated in a retrospective analysis of pooled data from 297 patients who underwent regional or general anesthesia (Heller et al., 2008). Patients received 53 ± 30/2.65 ± 1.5 mg cafedrine/theodrenaline.kg^−1^ when the systolic blood pressure dropped below 80% of the baseline value. A rapid increase in blood pressure (maximum mean arterial pressure (MAP) increase 9 ± 4 min after drug administration) was observed. The increase in MAP after 5 min was 11 ± 14 mmHg and after 10 min 14 ± 16 mmHg. The ED_50_ of cafedrine/theodrenaline was 1.49/0.075 mg.kg^−1^ to achieve a 10% increase in MAP after 5 min, and 0.53/0.027 mg.kg^−1^ to achieve a 10% increase after 10 min. Analysis of data from different dose groups in the range from 0.31 ± 0.07 mg/kg to 1.25 ± 0.44 suggested a direct dose-response. Cafedrine/theodrenaline's effect on mean arterial pressure was significantly less substantial in patients with heart failure and in patients with a higher MAP at baseline. Furthermore, male patients showed a less substantial effect than females. The authors suggested that a higher intravascular fluid volume in women might explain this difference (Heller et al., 2008).

The effects described above are supported by the results of a more recent study which demonstrated an increase in MAP of 11 ± 16 mmHg 5 min after administration of 1.25 ± 1.0 mg/64 ± 50 μg cafedrine/theodrenaline.kg^−1^ in a separate patient population undergoing regional and general anesthesia. Patients were included after a drop in MAP ≥ 5% (Heller et al., 2015). The highest MAP was reached 17.4 ± 9.0 min after administration of an average dose of 1.27 ± 1.0 mg/64 ± 50 μg cafedrine/theodrenaline.kg^−1^. The same authors confirmed a more rapid 10% MAP increase in women and a faster increase to maximum MAP in patients without heart failure. Patients without heart failure also required less cafedrine/theodrenaline to reach a similar magnitude of MAP increase: 1.16 ± 0.77 mg/58.0 ± 38.5 μg cafedrine/theodrenaline.kg^−1^ to achieve an increase of 14 ± 14 mmHg vs. 1.78 ± 1.67 mg/89.0 ± 83.5 μg cafedrine/theodrenaline.kg^−1^ to achieve an increase of 14 ± 16 mmHg.

In a study involving 20 patients undergoing epidural anesthesia, Seitz et al. showed that MAP increased by 43.0% after administration of 100/5 mg cafedrine/theodrenaline (Seitz et al., 1985).

### Shock

Animal data have demonstrated that cafedrine/theodrenaline causes positive inotropy in dogs with cardiogenic shock, including an increase in MAP, heart rate, pressure in the left ventricle, and blood flow in *A. femoralis* and *R. circumflexus* (Hahn et al., 1985). Two older (German) publications recommend the use of cafedrine/theodrenaline in patients with non-hemorrhagic shock (Böhmert, 1969; Bartels et al., 1978), one of which specifies that cafedrine/theodrenaline leads to a stronger increase of systolic blood pressure compared to the diastolic pressure in normovolaemic patients and therefore to a larger amplitude. The increased blood pressure is mainly due to an increased stroke volume. The previously-increased systemic vascular resistance decreased after cafedrine/theodrenaline administration (Böhmert, 1969).

### Safety profile

When cafedrine/theodrenaline was launched in Germany in the 1960s, safety standards for medicinal products were very different from today.

Cafedrine/theodrenaline are theophylline-derivates. There are studies showing that theophylline may lead to tachycardia and arrhythmias (Bittar and Friedman, 1991; Chazan et al., 1995). In the course of developing cafedrine/theodrenaline, a number of theophylline-derivates were searched for candidates that combine the beneficial cardiovascular effect and a better side effect profile. The derivates' circulatory effects indeed differ from theophylline (Daweke and Oberdorf, 1958; Schlepper and Witzleb, 1962). In general, the impact of cafedrine/theodrenaline differs from those of the individual substances (Sternitzke et al., 1984). A review from 2006 states that tachycardia and arrhythmias are hardly ever observed with cafedrine/theodrenaline (Koch and Wenzel, 2006).

After more than 50 years of use and ~50–100 million patients treated with the drug, cafedrine/theodrenaline has an anecdotally good safety record (ratiopharm GmbH, 2013), but there are few safety data from clinical studies.

### Renal function

While theodrenaline alone showed an antidiuretic effect in rats, cafedrine and a 20:1 combination of cafedrine and theodrenaline exhibited diuretic effects (Sakai et al., 1972a). The authors of a small study involving 12 patients (normotensive or hypotensive, normal, or impaired kidney function) concluded that cafedrine/theodrenaline shows a favorable renal profile. Based on theoretical considerations, they concluded that its renal profile is especially favorable compared with vasopressor sympathomimetics (Bihler et al., 1972). A small increase in both glomerular filtration rate and renal blood flow occurred in their study, but both were non-significant (Bihler et al., 1972).

### Obstetrics

Cafedrine/theodrenaline is regularly used in obstetric anesthesia (Marcus et al., 2011). Pregnant women are at a particular risk of hypotension during anesthesia (general and regional) due to reduced cardiovascular reserve (Koch and Knoth, 2006), but preservation of uteroplacental perfusion is important in order to avoid fetal acidosis (Koch and Knoth, 2006).

Experiments in pregnant sheep without hypotension demonstrated an increase in maternal MAP of maximum 14.2 ± 3.2% for 6 min after administration of a fixed dose of 100/5 mg cafedrine/theodrenaline (Gogarten et al., 2004). The authors of this animal study highlight possible differences between species and suggest very careful dosing to avoid a decrease in uteroplacental perfusion.

In one retrospective study involving parturients undergoing spinal anesthesia for cesarean section, 117 out of 173 (68%) patients exhibited a drop in blood pressure. Fifty-six patients whose systolic blood pressure remained within defined limits, i.e., ≥120 mmHg or >80% of baseline blood pressure, were assigned to the non-treatment group and were compared with the hypotensive patients treated with cafedrine/theodrenaline (Clemens et al., 2010). In the treated group, an increase in mean systolic blood pressure of 8.6 mmHg 1 min after administration of a mean of 43 ± 11/2.2 ± 0.6 mg cafedrine/theodrenaline was observed. Comparably, systolic blood pressure increased (mean 21.3 mmHg) and no negative impact on umbilical cord pH or APGAR score was observed.

The German Society of Anesthesiology and Intensive Care Medicine and the German Association of Gynecology and Obstetrics recommend the use of cafedrine/theodrenaline, as an alternative to phenylephrine, in hypotensive states associated with anesthesia for Cesarean section (Durchführung von Analgesie- und Anästhesieverfahren in der Geburtshilfe, 2009). However, this recommendation is not based on randomized clinical studies.

### Alternative routes of administration

A study by Schleusing and Bartsch compared intravenous and intramuscular administration of 200/10 mg cafedrine/theodrenaline in 25 healthy volunteers and 26 patients with cardiovascular disease. When compared with the intravenous route, intramuscular administration induced a more moderate and delayed, but also a longer lasting increase in blood pressure (Schleusing and Bartsch, 1963). This difference between intramuscular and intravenous administration was confirmed in a study involving 252 patients with intra- or post-operative hypotension (Eichler and Stephan, 1964).

Measurement of cardiovascular parameters before and 30 min after oral administration of 100/5 mg cafedrine/theodrenaline and measurement of orthostatic cardiovascular changes after 4 weeks of daily oral cafedrine/theodrenaline administration were conducted in a study involving 20 children with orthostatic hypotension (Hoffmann and Sternitzke, 1977). Use of cafedrine/theodrenaline resulted in constriction of the leg veins and, as a result, a 37% reduction in venous pooling (Hoffmann and Sternitzke, 1977). In combination with increased venous return and increased contractility, this led to increased cardiac output and decreased systemic vascular resistance (Hoffmann and Sternitzke, 1977).

## Assessment of data quality

Many of the data cited in this review were published in the 1960s and scientific standards have changed substantially since then. However, data relating to the use of cafedrine/theodrenaline in the management of hypotension treatment are scarce, and these studies have been included to give a comprehensive summary of the current state of knowledge.

## Future research

Future areas of research on cafedrine/theodrenaline could include: (a) a more comprehensive description of the pharmacokinetics (e.g., determination of how long the individual components stay in the body); (b) further elucidation of the mechanism of action of each of the components and their cleavage products; (c) characterization of the inotropic, bathmotropic, and chronotropic effects of cafedrine/theodrenaline (possibly involving the use of isolated Langendorff preparations); (d) characterization of the temporal relationship between cafedrine/theodrenaline administration and vasoconstriction or vasodilatation in isolated venous and arterial vessel preparations; and (e) comparison of the effects of cafedrine/theodrenaline and alternative drugs on blood pressure, heart rate, and cardiac output in a clinical context (e.g., head-to-head comparisons with internationally used substances, such as ephedrine and phenylephrine). A multicenter, non-interventional study of cafedrine/theodrenaline vs. ephedrine for the treatment of perioperative hypotensive states is currently ongoing, and is registered in ClinicalTrials.gov (NCT02893241) and in the German Clinical Trials Register (DRKS-ID: DRKS00010740).

## Cafedrine/theodrenaline in comparison with other pressor agents

In this review article we focus on cafedrine/theodrenaline, but there are other antihypotensive agents available. Phenylephrine stimulates predominantly the α-receptors and is a strong vasopressor which leads to an increase in blood pressure and a reflex bradycardia. The vascular resistance is increased, which in turn leads to a decrease of the cardiac output (Sintetica, 2015). Ephedrine stimulates both, α- and β-receptors, has an inotropic and chronotropic effect and therefore increases the cardiac output (Sintetica, 2014). In addition, ephedrine stimulates the heart rate (Loubert, 2012).

Unfortunately, there is a lack of high quality head-to-head studies between cafedrine/theodrenaline and other pressor agents. However, one study from 1985 compared equipotent doses of cafedrine/theodrenaline, etilefrine, ephedrine, norfenefrin, and amezinium in 50 anesthetized patients (Muller et al., 1985). The authors concluded that vasopressors that stimulate predominantly the α-receptors, such as norfenefrin, may lead to an unwanted high increase of the systemic vascular resistance and a subsequent decrease of cardiac output. Predominantly betamimetic substances may display a delayed increase of blood pressure. The authors recommended the use of α- and β-receptor-stimulating substances, such as ephedrine and cafedrine/theodrenaline, which successively provide both venoconstriction and cardiac stimulation.

## Conclusion and authors' personal opinion

Cafedrine/theodrenaline leads to a rapid increase in blood pressure and the pharmacologic properties exhibit many of the characteristics of an ideal antihypotensive agent. As outlined in section “Effects of Cafedrine and Theodrenaline in Combination,” the increase in blood pressure is mainly due to increased inotropy, cardiac preload, stroke volume, and cardiac output, while the systemic vascular resistance remains largely unchanged. This is an advantage compared to drugs which act predominantly via activation of α_1_-adrenoceptors. In addition, the heart rate remains mostly unchanged. This may be advantageous compared with drugs that exhibit a pronounced activation of β_1_-receptors, since tachycardia is an unwanted side effect with respect to myocardial oxygen consumption. The duration of the effect is long-lasting possibly due to the proposed combination of direct and indirect sympathomimetic properties and simultaneous PDE-inhibition (Figure 1). The long-lasting effect enables comfortable drug handling, because repeated bolus administrations or the use of syringe pumps can be avoided.

Also, this drug combination has a very good safety record, as it has been used widely in Germany since the 1960s. However, it should be noted that there are only few safety data from controlled trials available.

## Author contributions

BB, TC, and LE have all substantially contributed to the conception of the manuscript, revised it critically, approved it before submission and agree to be accountable for all aspects of the work.

### Conflict of interest statement

LE reports honoraria for lectures from Baxter GmbH, Fresenius GmbH, Grunenthal GmbH and ratiopharm GmbH (the manufacturer of Akrinor®). BB reports personal fees from ratiopharm GmbH, Pulsion Medical Systems, Air Liquide International, Orion Pharma, MSD, GE Healthcare, CNSystems, Edwards Life Sciences, CSL Behring, and Abbvie outside the submitted work. TC receives support for his research by the German Network for Heart Research (DZHK, partner site Hamburg/Kiel/Lübeck), ratiopharm GmbH, Ulm, Germany and Xention Ltd, Cambridge, UK. Furthermore he received a speaker honorary from ratiopharm GmbH.
