# Supplementary material for: Cafedrine/Theodrenaline (20:1) Is an Established Alternative for the Management of Arterial Hypotension in Germany—a Review Based on a Systematic Literature Search
Source: Front Pharmacol. 2017 Feb 21;8:68. doi: 10.3389/fphar.2017.00068 (PMC5318387; doi:10.3389/fphar.2017.00068)
Supplement: Supplementary file 1 [file Table1.docx]

**Cafedrine/Theodrenaline (20:1) is an Established Alternative for the Management of Arterial Hypotension in Germany - a Review Based on a Systematic Literature Search**

Berthold Bein^1^, Torsten Christ^2^, Leopold H. J. Eberhart*^3^

***Correspondence:** Leopold H. J. Eberhart: [eberhart@staff.uni-marburg.de](mailto:eberhart@staff.uni-marburg.de)

**Supplemental material. Table 1**

Overview of study details demonstrating the cardiovascular effects of cafedrine/theodrenaline. Please note that the standard of scientific publications has changed since some of these studies were published.

| **Reference** | **Patient population** | **Study design details** | **Investigated outcomes** | | **Results** |
| --- | --- | --- | --- | --- | --- |
| Fischer and Weis, 1965(1) | Healthy male subjects | Injection of 2.5 mg.kg^-1^ /125 µg.kg^-1^ or 1.25 mg.kg^-1/^ 63 µg.kg^-1^ cafedrine/theodrenaline (n = 12 each)  Repeated injection of 1.25 mg.kg^-1^/63 µg.kg^-1^ cafedrine/theodrenaline (n = 5)  Injection of 1.25 mg.kg^-1^ /63 µg.kg^-1^ cafedrine/theodrenaline during ganglion blockade (n = 4) | - Blood pressure - Heart rate - Circulatory parameters - Spirometry | | - 2.5 mg group: Increase in blood pressure (maximum increase 2 minutes after injection, 30% increase in MAP) - 1.25 mg group: Increase in blood pressure (maximum increase 2 minutes after injection, 11% increase in mean blood pressure) - Decrease in heart rate of 17 and 13 beats per minute (2.5 and 1.25 mg group, respectively) - Increase in stroke volume and cardiac output in both groups - Repeated injection of cafedrine/theodrenaline led to a small increase in efficacy - Cafedrine/theodrenaline injection during ganglion blockade led to typical circulatory changes, similar to the results described above - Increased respiratory rate and tidal volume |
| Hahn, Sternitzke et al., 1985(2) | Anaesthetized dogs with experimental cardiogenic shock (2 test series with 9 and 10 dogs) | Animal study. Induction of cardiogenic shock by occlusion of the coronary arteries  Administration of 5 mg.kg^-1^ /250 µg.kg^-1^ cafedrine/theodrenaline | | - Cardiovascular parameters - Oxygen consumption | - Cardiogenic shock led to decreased MAP and heart rate which could be reversed by increased inotropy following cafedrine/theodrenaline administration - Increased oxygen consumption after cafedrine/theodrenaline administration (from 4.8 ml.min^-1^ per 100 g to 11.0  ml.min^-1^ per 100 g). - Increased blood flow in right circumflex coronary artery (+181%) - Sufficient net oxygenation |
| Heller and Grosser, 1974(3) | Patients with acute cardiac infarction without shock (n = 14) | Measurements at baseline and 2, 5, 10, 20, 30 and 40 minutes post-administration of 200 mg/10 mg cafedrine/theodrenaline | | - Arterial blood pressure - Pulmonary artery pressure - Cardiac output | - Significant increase in arterial pressure in all patients (+14.4 mmHg [mean], maximum after 5 minutes, p = 0.0005) - Similar observations in mean pulmonary artery pressure - No substantial increase after 2 and 5 minutes, maximum increase of cardiac output after 10 minutes - Favorable mode of action for patients with myocardial infarction |
| Heller, Radke et al., 2008(4) | Patients who underwent regional or general anesthesia (n = 297) | Retrospective analysis of anesthesia protocols (mean dosis: 120/6 mg cafedrine/theodrenaline) | | - MAP change after 5 and 10 minutes - Time to maximum MAP - Change in heart rate - Effect of gender, β-blocker administration and heart failure on the effects of cafedrine/theodrenaline | - MAP increase of 11 ± 14 and 14 ± 16 mmHg after 5 and 10 minutes - MAP maximum after 9 ± 4 minutes - Heart rate increased by 2 ± 10/minute - Male gender, use of β-blockers and heart failure reduced the effect of cafedrine/theodrenaline |
| Heller, Heger et al., 2015(5) | Patients who underwent regional or general anesthesia (n = 353) | Retrospective analysis of anesthesia protocols  20:1 cafedrine/theodrenaline administration (mean 1.3 mg.kg^-1^/65 µg.kg^-1^ (women) and 1.2 mg.kg^-^1/60 µg.kg^-1^ (men)) | | - Time to 10% MAP increase - Potential factors influencing the effects of cafedrine/theodrenaline | - 10% MAP increase after 7.2 ± 4.6 (women) and 8.6 ± 6.3 (men) minutes (p = 0.018) - Patients with heart failure require higher doses of cafedrine/theodrenaline to increase MAP by 14 ± 16 mmHg at 15 minutes (1.78 ± 1.67 mg.kg^-1^ [cafedrine], 89.0 ± 83.5 µg/kg [theodreanline] vs. 1.16 ± 0.77 mg/kg [cafedrine], 58.0 ± 38.5 µg/kg [theodrenaline] ), p = 0.005 - Prolonged time to 10% MAP increase with β-blocker administration (9.0 ± 7.0 vs 7.3 ± 4.3 minutes, p = 0.008) - No clinically significant change in heart rate |
| Muller, Brahler et al., 1985(6) | Anesthetized subjects with induced hypotension (n = 50) | Administration of equipotent amounts of cafedrine/theodrenaline (450 µg.kg^-1^/22.5 µg.kg^-1^), etilefrine, ephedrine, norfenefrin, amezinium | | - Hemodynamic parameters | - Cafedrine/theodrenaline increased blood pressure until up to 5 minutes after administration - Increased myocardial contractility, cardiac index and preload |
| Sakai, Shioya et al., 1972(7) | Mongrel dogs (n = 57) and albino rats (n = 50) | Administration of 250 µg.kg^-1^ theodrenaline (intra-arterially or intravenously), alone or in combination with cafedrine in various ratios (1:5, 1:10, 1:20 and 1:50) | | - Diuretic effects and renal circulation | - 250 µg theodrenaline increased renal vascular resistance in dogs; this was inhibited by a 10:1 and 20:1 mixture of cafedrine:theodrenaline - Theodrenaline’s renal vasoconstrictor effect in dogs was abolished when cafedrine was administered in a 20:1 ratio - Theodrenaline showed an anti-diuretic effect in rats; cafedrine and a 20:1 combination of cafedrine:theodrenaline induced diuresis |
| Schieffer, Heinz et al., 1971(8) | Healthy subjects (n = 15) and patients with mitral valve disease and stenosis (stage II; not decompensated) (n = 8) | Measurement of pressures in the aorta, brachial artery, pulmonary artery and – in 6 patients – right ventricle  Administration of 100 mg/5 mg cafedrine/theodrenaline | | - Changes in the circulatory system - Changes in cardiac parameters | - Immediate systolic and MAP increase (systolic +28 mmHg, diastolic +7 mmHg in healthy subjects, similar in patients) due to increased cardiac output - Heart rate and systemic vascular resistance slightly decreased - Presumably increased preload |
| Schlepper and Witzleb 1962(9) | Anesthetized dogs (n = 9) | Animal study. Administration of 4 mg.kg^-1^/200 µg.kg^-1^ or <4 mg.kg^-1^/200 µg.kg^-1^ cafedrine/theodrenaline in 2 groups | | - Oxygen consumption - Coronary blood flow - Blood pressure - Heart rate | - Increased oxygen consumption (+123% [mean] in the 4 mg/kg group) - Increased coronary blood flow; proportionally higher than increase in oxygen consumption - Increased systolic blood pressure in 9 out of 10 cases. Mean blood pressure increased 25 % in the 4 m.kg^-1^ group - Increased heart rate in 8 out of 10 cases (14% mean increase in the 4 mg.kg^-1^ group) |
| Schleusing and Bartsch, 1963(10) | Healthy subjects (n = 25) and patients with myocardial damage (n = 23) or hypertension (n = 3) | Intramuscular cafedrine/theodrenaline administration (200 mg/10 mg) to 31 subjects and intravenous administration to 20 subjects | | - Change in blood pressure - Time to effect and duration - Heart rate - Effects on heart of intramuscular and intravenous administration | - Substantial increase in systolic blood pressure - Delayed effect after intramuscular administration, duration 60 – 90 minutes. Effect more rapid and substantial but of shorter duration after intravenous administration - (Mild) decrease in heart rate - More substantial response in patients with myocardial damage and hypotension |
| Sternitzke, Schieffer et al., 1975(11) | Healthy subjects (n = 10) | Administration of 100 mg/5 mg cafedrine/theodrenaline, followed by administration of the β-blocker propranolol, followed by re-administration of 100 mg/5 mg cafedrine/theodrenaline | | - Cardiovascular effects - Oxygen uptake - Measurement of arterial pressures in the aorta and pulmonary artery | - Cafedrine/theodrenaline showed β-receptor stimulation - 60% increase in stroke volume and 36% increase in cardiac output per minute - Cafedrine/theodrenaline led to an increase in oxygen uptake before and after propranolol (28% and 15%, respectively) - Cafedrine/theodrenaline led to a 16% increase in pulmonary artery pressure before propranolol and to a slight increase of 1 mmHg after propranolol administration |
| Sternitzke, Schieffer et al., 1976(12) | Healthy subjects | Administration of 100 mg/5 mg cafedrine/theodrenaline, followed by administration of the β-blocker, propranolol, followed by re-administration of 100 mg/5 mg cafedrine/theodrenaline | | - Cardiovascular effects | - β-receptor stimulation - 15% decrease in heart rate - No increase in systemic vascular resistance - 22% increase in systolic blood pressure with cafedrine/theodrenaline before propranolol, 58% increase in stroke volume/time after propranolol - Decrease in heart rate, especially after the second cafedrine/theodrenaline administration |
| Sternitzke, Schieffer et al., 1984(13) | Healthy subjects (n = 25) | Administration of 200 mg and 300 mg cafedrine (n = 12 and 11 examinations), 5 mg theodrenaline (n = 16) or a 20:1 combination of both (100 mg/5 mg cafedrine/theodrenaline, n = 9) | | - Hemodynamic effects of the individual components in comparison with the combination | - Theodrenaline: increase in blood pressure from first minute onwards, effect decreasing until 20 minutes after administration, 21% increase in systemic vascular resistance - Cafedrine: delayed increase in blood pressure, reaching its maximum at 20 and 60 minutes (for 200 and 300 mg, respectively), no effect on systemic vascular resistance - Combination: increased blood pressure from first minute onwards, prolonged effect. Mostly no effect on systemic vascular resistance, or tendency to decrease |

MAP, mean arterial pressure

**References**

1. Fischer F, Weis KH. [Experimental Circulatory Tests and Clinical Experiences with 2 Theophylline Derivatives]. *Der Anaesthesist* (1965) **14**:147-53. Epub 1965/05/01. PubMed PMID: 14303298.

2. Hahn N, Sternitzke N, Malotki B, Raqué B, Eichelkraut W, Forneck G. Der Einfluß von Akrinor auf die Hämodynamik und die Myokarddurchblutung bei partiell ischämiegeschädigtem Herzen (kardiogener Schock). Akute Versuche am narkotisierten Hund. *Herz Kreislauf* (1985) **9**:464.

3. Heller A, Grosser KD. [Hemodynamics in patients with myocardial infarct following intravenous administration of Akrinor]. *Die Medizinische Welt* (1974) **25**(45):1890-2. PubMed PMID: 4437380.

4. Heller A, Radke J, Koch T. Proof of efficacy and dose-response relationship of Akrinor® in patients during general and regional anaesthesia. *Anästhesiologie & Intensivmedizin* (2008) **49**:308-17.

5. Heller AR, Heger J, Gama de Abreu M, Muller MP. Cafedrine/theodrenaline in anaesthesia: Influencing factors in restoring arterial blood pressure. *Der Anaesthesist* (2015) **64**(3):190-6. Epub 2015/03/12. doi: 10.1007/s00101-015-0005-y. PubMed PMID: 25757552; PubMed Central PMCID: PMC4383809.

6. Muller H, Brahler A, Borner U, Boldt J, Stoyanov M, Hempelmann G. [Hemodynamic effects following bolus administration of different vasopressive agents for blood pressure stabilization during peridural anesthesia]. *Regional-Anaesthesie* (1985) **8**(3):43-9. PubMed PMID: 2863856.

7. Sakai K, Shioya A, Hashimoto K. Effect of combining noradrenalinetheophylline and norephedrinetheophylline in various ratios on renal circulation. *Arzneimittelforschung* (1972) **22**(4):698-701. Epub 1972/04/01. PubMed PMID: 5068249.

8. Schieffer H, Heinz H, Sternitzke N, Bette L. [Effect of Akrinor on the cardiac and circulatory dynamics as well as on pulmonary circulation in patients with and without heart diseases]. *Verh Dtsch Ges Inn Med* (1971) **77**:948-52. Epub 1971/01/01. PubMed PMID: 5156093.

9. Schlepper M, Witzleb E. Coronardurchblutung und O2-Verbrauch des Warmblüterherzens unter dem Einfluß eines Kreislaufanalepticums mit neuartigem Wirkungscharakter. *Arzneimittel-Forschung [Drug Research]* (1962) **12**:841-3.

10. Schleusing G, Bartsch C. Die Wirkung von synthetischen Theophyllin-Derivaten mit kreislaufwirksamen Molekülgruppen auf das Verhalten von Blutdruck und Pulsfrequenz sowie auf das Elektrokardiogramm bei Kreislaufgesunden und Kreislaufkranken. *Arzneimittel-Forschung* (1963) **13**:470-74.

11. Sternitzke N, Schieffer H, Bette L. [Effect of Akrinor on cardiovascular-dynamics before and after blockade of adrenergic beta-receptors by propranolol]. *Zeitschrift für Kardiologie* (1975) **64**(5):419-30. Epub 1975/05/01. PubMed PMID: 1189536.

12. Sternitzke N, Schieffer H, Hoffmann W, Bette L. [Modification of the cardiovascular-dynamic effect of Akrinor following the blockade of adrenergic beta receptors with propranolol]. *Verhandlungen der Deutschen Gesellschaft fur Innere Medizin* (1976) **82 Pt 2**:1132-5. PubMed PMID: 197729.

13. Sternitzke N, Schieffer H, Rettig G, Bette L. Die Beeinflussung der Herz-Kreislauf-Dynamik durch die Theophyllin-Verbindung Cafedrin und Theodrenalin sowie durch ihre Kombination. *Herz Kreislauf* (1984) **8**:401-12.
